# Bridging developmental and statistical approaches to variation and evolution

**DOI:** 10.1073/pnas.2529820123

**Published:** 2026-03-11

**Authors:** Lisandro Milocco, Tobias Uller

**Affiliations:** ^a^Department of Zoology, SciLifeLab, Stockholm University, Stockholm 106 91, Sweden; ^b^Department of Biology, Lund University, Lund 223 62, Sweden

**Keywords:** evolvability, quantitative genetics, evo-devo, dynamical systems

## Abstract

Phenotypic variation is the raw material for evolutionary diversification and adaptation. However, a critical gap remains in evolutionary theory between developmental and statistical representations of phenotypic variation, limiting our ability to understand and predict evolutionary change. In this paper, we close this gap by establishing a formal bridge between developmental and statistical accounts of phenotypic variation. Representing development as a dynamical system, we derive explicit relationships between perturbations to developmental systems and quantitative-genetic parameters. Through this framework, we obtain two important results. First, we show that the full developmental trajectory contains information that can improve the estimation of statistical parameters relevant to evolution. Second, we explain how different sources of variation—genetic, environmental, and stochastic—shape the distribution of phenotypic variation. This reveals conditions under which covariance matrices are expected to align, offering a developmental explanation for statistical patterns of phenotypic variation at both micro- and macroevolutionary scales. These findings advance our understanding of how developmental processes structure phenotypic variation, shape evolutionary dynamics, and influence evolvability.

## Significance

Evolution depends on variation, but the developmental processes that generate this variation have often been overlooked in evolutionary biology, which traditionally emphasizes statistical descriptions at the population level. Here, we present a framework that formally links these statistical descriptions to the underlying processes that generate phenotypic variation. We show that this link allows us to improve estimates of key evolutionary quantities and can help clarify empirical patterns that are surprising when analyzed from a purely population-level perspective. This work shows that the integration of population and developmental-level understandings of variation not only advances theoretical insight but also enables the construction of concrete mathematical tools to better understand and predict evolutionary change, an increasingly urgent goal amid rapid environmental change.

An essential prerequisite for evolution by natural selection is variation among individuals in phenotypic traits that affect fitness (1, 2). Not only the amount of variation, but also the combinations of traits that are common, determine the directions in which evolution can proceed. Understanding the structure of variation is therefore central to understanding evolution.

The dominant framework for studying phenotypic variation in evolutionary biology is quantitative genetics. This framework decomposes phenotypic (co)variance into genetic and environmental components, as well as their interactions, with genetic (co)variance further partitioned into additive and nonadditive contributions (3, 4). In practice, most evolutionary applications emphasize additive genetic (co)variance because it predicts the response to selection (5) and can quantify a population’s capacity to evolve (evolvability) (2). This framework also underpins empirical methods such as quantitative trait locus (QTL) mapping and genome-wide association studies (GWAS) (6). Its power lies in its generality: It can be applied across species and traits without detailed knowledge of the mechanisms generating variation.

Yet this generality comes at a cost. Quantitative genetics treats the production of phenotypes through development as a black box (7). As a result, it offers a descriptive and correlational view of variation but does not explain how phenotypes arise, or why some kinds of variation are common while others are rare. In contrast, evolutionary developmental biology (evo-devo) and systems biology focus on the generative process itself. By studying the dynamic interactions among genes, cells, and environments, these frameworks explain how phenotypic variation emerges through ontogeny (8–10). This mechanistic perspective has revealed principles that purely statistical models cannot capture, including the origins of novel traits (7, 11) and the ways in which genetic and environmental perturbations can bias phenotypic outcomes in similar directions (12, 13).

Integrating developmental insights into the statistical machinery of quantitative genetics has long been a goal (14–19). One approach is to embed detailed mechanistic models of development directly into quantitative genetic frameworks by treating the parameters of these models as quantitative traits (20–22). While informative, this strategy has limited applicability because detailed mechanistic models are rare.

A more general path is to identify broad properties of generative processes that can be translated into the statistical formalism of quantitative genetics. Such properties would allow developmental knowledge to inform statistical models even without a detailed mechanistic representation, combining the explanatory depth of development with the broad applicability of quantitative genetics.

Here, we pursue this integration. Representing development as a dynamical system (9, 23) and applying the formalism of sensitivity vectors (13, 24), we derive general expressions that link developmental dynamics to key statistical quantities in quantitative genetics. These expressions are not tied to any specific model of development but instead capture general features that arise from treating development as a dynamical system and translating them into the language of quantitative genetics.

This connection yields two major advances. First, it provides a principled way to use developmental dynamics, captured in time-series data, to improve the estimation of statistical parameters that underpin evolutionary predictions. Second, it reveals simple developmental explanations for statistical patterns of phenotypic variation-both within and between populations—that would otherwise appear unexpected and are central to evolutionary inference. Specifically, the framework identifies the conditions under which (co)variance matrices generated by different sources of variation—such as genetic, environmental, or noise—are expected to be proportional (25–28), and can help explain why directions of standing variation within populations may align with long-term phenotypic divergence (26, 28–30). Together, these advances show how developmental insights can deepen our understanding of phenotypic variation and expand the predictive scope of quantitative genetics.

## Results

The results are presented in four parts. First, we describe development as a dynamical system and introduce sensitivity vectors from prior work (13). Second, we relate these sensitivities to classical quantitative-genetic parameters, such as the average effects of alleles. Third, we use this connection to improve estimates of average genetic effects over developmental time. Finally, we show how sensitivity vectors reveal how different sources of variation shape phenotypic covariance matrices and explain patterns of variation that appear surprising from a statistical, quantitative genetics approach.

### Development as a Dynamical System and Sensitivity Vectors.

To integrate developmental biology and quantitative genetic theory, we need a general representation of development that captures fundamental properties of generative processes without being tied to specific biological systems. Accordingly, and following previous work (8, 9, 13, 14, 23), we model development as a dynamical system. This representation captures the generation of the phenotype over developmental time by rules that determine how the current phenotypic state gives rise to future states via regulatory, biochemical, or mechanical interactions among the components of the developmental system. Mathematically, these rules can be expressed as systems of differential equations that generate a developmental trajectory (9, 13, 23). Using bold symbols to denote vectors, we write this general representation of development as

[1] $$\begin{array}{c} \dot{x}=f\left(t,x,\lambda \right),\;x\left(t_{0}\right)=x_{0}, \end{array}$$

where $x={\left(x_{1},x_{2},\cdots ,x_{n}\right)}^{T}\in R^{n}$ is the state vector of the system and ^T^ denotes the transpose. Each component $x_{i}$ represents a phenotypic variable (e.g., the expression level of a gene), and together they describe the full developmental state of the organism. The vector $\dot{x}$ denotes their time derivative, and t is developmental time. The function f governs the state dynamics and is assumed to be smooth. The initial conditions are given by $x_{0}$, and $\lambda ={\left(\lambda_{1},\lambda_{2},\cdots ,\lambda_{p}\right)}^{T}\in R^{p}$ is a vector of p developmental parameters of genetic or environmental origin. These may include, for example, the strength of activation or inhibition of a transcription factor, or the diffusion rate of a signaling molecule. For any choice of λ, smoothness ensures that the system has a unique solution. In particular, we define the reference parameters $\lambda^{*}$ which specify the reference (wildtype) developmental parameters. The corresponding trajectory, $x(t,\lambda^{*})$, is the unique solution of Eq. **1** with parameters $\lambda^{*}$ and initial condition $x_{0}$.

To analyze how small changes in the developmental parameters affect the system, we employ the formalism of sensitivity vectors (see details in ref. 13). Let $\lambda_{k}$ be the k-th developmental parameter with reference value $\lambda_{k}^{*}$. The corresponding sensitivity vector is defined as

[2] $$\begin{array}{c} s_{k}\left(t\right)=\frac{\partial x(t,\lambda )}{\partial \lambda_{k}}{|}_{\lambda =\lambda^{*}}. \end{array}$$

This vector captures how all phenotypic variables respond to an infinitesimal change in $\lambda_{k}$ from its reference value $\lambda_{k}^{*}$. The sensitivity vector defined in Eq. **2** satisfies the differential equation

[3] $$\begin{array}{cc} {\dot{s}}_{k}\left(t\right) & =A\left(t,\lambda^{*}\right)\;s_{k}\left(t\right)+b_{k}\left(t,\lambda^{*}\right),\;s_{k}\left(t_{0}\right)=0, \end{array}$$

with

$$\begin{array}{c} A\left(t,\lambda^{*}\right)=\frac{\partial f(t,x,\lambda )}{\partial x}{|}_{x=x(t,\lambda^{*})},b_{k}\left(t,\lambda^{*}\right)=\frac{\partial f(t,x,\lambda )}{\partial \lambda_{k}}{|}_{x=x(t,\lambda^{*})} \end{array}$$

Here, $A(t,\lambda^{*})$ is the Jacobian matrix of the system, and $b_{k}\left(t,\lambda^{*}\right)$ captures the direct dependence of f on the parameter $\lambda_{k}$. Both depend on t because they generally change over developmental time. Provided that f is known, these quantities can be computed and numerical integration of Eq. **3** yields $s_{k}\left(t\right)$.

As demonstrated in previous work (13), sensitivity vectors are a powerful tool for characterizing phenotypic variation arising from multiple sources, thereby revealing directions of potential evolutionary change. However, in most cases the explicit form of the developmental function f is not available, precluding an analytical evaluation of $s_{k}\left(t\right)$ using Eq. **3**. In the next section, we show how sensitivity vectors can be estimated directly from observational data on developmental trajectories, and how these estimates relate to classical quantitative-genetic parameters.

### Proportionality Between Sensitivities and Average Allelic Effects.

In this section, we establish a formal equivalence between sensitivities and the average effects of alleles, a central concept in quantitative genetics. Average effects quantify how much, on average, an allele influences a trait when inherited. Methods such as GWAS are designed to identify genomic regions with nonzero average effects, and polygenic scores rely on summing these effects across loci to predict complex traits, such as disease risk (6). Most importantly for evolutionary biology, the variance of the sum of average effects across individuals in a population is the additive genetic variance, a key determinant of evolvability (2) and a predictor of evolutionary response (5). In this way, average effects are a central statistic in evolutionary biology, and linking them to developmental dynamics is essential for unifying statistical and mechanistic views of phenotypic variation.

**Fig. 1.**
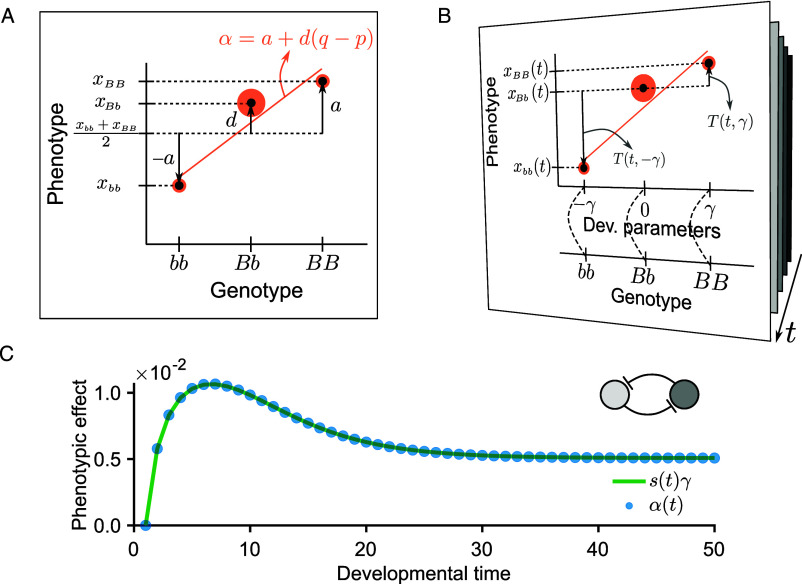
Proportionality between average effect, α(t), and sensitivity vector, s(t). (*A*) In the classical single-locus, single-trait model, genotypic values ($x_{\mathrm{bb}}$, $x_{\mathrm{Bb}}$, $x_{\mathrm{BB}}$) are expressed in terms of additive (a) and dominance (d) values. Together with population frequencies (shown as orange areas), these determine the slope of the weighted linear regression used to compute α (orange line). (*B*) The sensitivity vector formalism expresses the genotype–phenotype map through developmental parameters shaping phenotypic trajectories over developmental time (arrow). We express the values of homozygotes as deviations from the heterozygote with a Taylor expansion using the sensitivities, $T\left(t,\gamma \right)=s\left(t\right)\gamma +s^{\left(1\right)}\left(t\right)\gamma^{2}/2+\ldots$, see Eq. **4**. (*C*) For small parameter changes γ, we showed that α(t)≈s(t)γ. We illustrate this using a bistable gene network model (*Inset*, *Top Right*), with a single locus affecting the developmental parameter $\lambda_{1}$ and a single trait (gene 1 expression; see *Materials and Methods*). s(t) was computed by numerically integrating Eq. **3**, and α(t) was estimated via the regression in Eq. **7**, using simulated trajectories from a population of 20 individuals (p=q=0.5).

We begin with the single-locus, single-trait case, following the standard introductory treatment of genetic effects in quantitative genetics texts (3, 4). Fig. 1*A* illustrates the classical quantitative-genetic representation of this system, in which we consider a biallelic locus with three possible genotypes: bb, Bb, and BB. For simplicity, we also consider a single developmental parameter in this section, extending the framework to multiple parameters, loci, and traits in later sections. Accordingly, in this section we adopt scalar notation—e.g., λ=λ, x=x and $s_{k}=s$.

A key insight is that allelic substitutions (i.e., replacing a b allele with a B allele) can be viewed as perturbations to the developmental system. This provides a way to study how small genetic changes propagate through development to affect the phenotype, as we formalize in terms of sensitivities and developmental parameters below.

The sensitivity s is linked to the developmental parameter λ. To relate this sensitivity to quantitative genetics, we decompose the genotype-to-phenotype map into two sequential steps: from genotype to developmental parameters and from developmental parameters to phenotype (Fig. 1*B*; see also refs. 9 and 21). For simplicity, we assume an additive mapping from genotype scores to developmental parameters. Consequently, any nonlinear effects, such as dominance, arise in the mapping from developmental parameters to phenotypic states (21, 31, 32).

Sensitivities are defined relative to a reference developmental trajectory. Let $\lambda_{\mathrm{bb}},\lambda_{\mathrm{Bb}}$ and $\lambda_{\mathrm{BB}}$ denote the values of the developmental parameter associated with genotypes bb, Bb, and BB, respectively, and define $\lambda_{\mathrm{Bb}}=\lambda^{*}$ as the reference parameter value, such that the heterozygote trajectory denoted by $x\left(t,\lambda_{\mathrm{Bb}}\right)=x_{\mathrm{Bb}}\left(t\right)$ is the reference trajectory. Moreover, let $\gamma =\lambda_{\mathrm{BB}}-\lambda_{\mathrm{Bb}}$ represent the effect on the developmental parameter of substituting a B allele with a b allele (Fig. 1*B*). Under our assumptions, it follows that $\lambda_{\mathrm{bb}}-\lambda_{\mathrm{Bb}}=-\gamma$.

In quantitative genetics, the values $x_{\mathrm{bb}}$, $x_{\mathrm{Bb}}$, and $x_{\mathrm{BB}}$ are called genotypic values (3, Ch. 4]. These deterministic quantities correspond to the expected phenotype of an organism with genotype bb, Bb, or BB, respectively, under a fixed environment and genetic background. That is, in the absence of variation in environment and genetic background, the genotypic values coincide with the actual phenotypes. To emphasize the fact that these genotypic values can be defined at each time-point of development, we express them as $x_{\mathrm{bb}}\left(t\right)$, $x_{\mathrm{Bb}}\left(t\right)$, and $x_{\mathrm{BB}}\left(t\right)$.

Using the sensitivity framework and under the assumptions detailed above, we can express these genotypic values by expanding the developmental trajectory in a Taylor series about the reference trajectory $x_{\mathrm{Bb}}$ at each developmental time t (13, 24). This expansion is valid for sufficiently small perturbations over a finite-time developmental window (see *SI Appendix*, Text for a formal justification):

$$\begin{array}{c} x\left(t,\lambda \right)=x_{\mathrm{Bb}}\left(t\right)+s\left(t\right)\left(\lambda -\lambda_{\mathrm{Bb}}\right)+s^{\left(1\right)}\left(t\right)\frac{{\left(\lambda -\lambda_{\mathrm{Bb}}\right)}^{2}}{2}+\ldots \end{array}$$

Here, $s^{\left(1\right)}\left(t\right)$ is the second-order derivative of x with respect to λ (equivalently, the derivative of s with respect to λ), evaluated at $\lambda_{\mathrm{Bb}}$, and the ellipsis indicate the additional higher-order terms of the expansion.

Using this expansion, the phenotypes of the homozygotes at each developmental time t can be expressed as (Fig. 1*B*):

[4] $$\begin{array}{cc} x_{\mathrm{bb}}\left(t\right) & =x\left(t,\lambda_{\mathrm{bb}}\right)=x_{\mathrm{Bb}}\left(t\right)-s\left(t\right)\gamma +s^{\left(1\right)}\left(t\right)\frac{\gamma^{2}}{2}+\ldots \\ x_{\mathrm{BB}}\left(t\right) & =x\left(t,\lambda_{\mathrm{BB}}\right)=x_{\mathrm{Bb}}\left(t\right)+s\left(t\right)\gamma +s^{\left(1\right)}\left(t\right)\frac{\gamma^{2}}{2}+\ldots \end{array}$$

In classical quantitative genetics, genotypic values are expressed in terms of additive and dominance values, denoted a and d, respectively. To highlight that these values can be calculated at each developmental time t, we write them here as a(t) and d(t). The additive value a(t) is defined as half the phenotypic difference between the homozygotes (3, Ch. 4], (Fig. 1):

[5] $$\begin{array}{c} a\left(t\right)=\frac{x_{\mathrm{BB}}\left(t\right)-x_{\mathrm{bb}}\left(t\right)}{2}=s\left(t\right)\gamma +\ldots \end{array}$$

Using our parameterization, only odd-order terms of the Taylor series contribute to a(t). In contrast, the dominance value d(t), defined as the deviation of the heterozygote from the mean of the homozygotes (3, Ch. 4; see Fig. 1), is given by

[6] $$\begin{array}{c} d\left(t\right)=x_{\mathrm{Bb}}\left(t\right)-\frac{x_{\mathrm{BB}}\left(t\right)+x_{\mathrm{bb}}\left(t\right)}{2}=-s^{\left(1\right)}\left(t\right)\frac{\gamma^{2}}{2}-\ldots \end{array}$$

Here, only the even-order terms contribute to d(t). The expression above reveals that both a(t) and d(t) can be expressed in terms of the sensitivities and further derivatives, all of which depend on the underlying developmental process f.

Additive and dominance values are properties of genotypes, but offspring inherit alleles rather than complete genotypes from parents. Consequently, quantitative genetics emphasizes the average effect of allelic substitutions. As mentioned above, this quantity is defined as the mean phenotypic difference between offspring inheriting allele B and those inheriting allele b (i.e., the effect of substituting a b allele with a B allele). Here, we denote the average effect by α(t) to highlight that it can be defined for each developmental time t. In contrast to the genotypic values a(t) and d(t) introduced above, α(t) is a population-level parameter that depends on the allelic frequencies, which we denote by p for allele b and q for allele B (with p+q=1).

The average effect can be estimated from the population, of size $N_{\mathrm{pob}}$, as the slope of the regression of phenotype on genotype score, as illustrated in Fig. 1*A*. This approach is grounded in Fisher’s decomposition, which we express in our notation with the heterozygote as the reference:

[7] $$\begin{array}{c} y(t)=\mu (t)\;1+z\;\alpha (t)+\delta (t), \end{array}$$

where y(t) is the column vector of phenotypic values for the $N_{\mathrm{pob}}$ individuals in the population at time t, each element corresponding to one individual and taking the value $x_{\mathrm{BB}}\left(t\right)$, $x_{\mathrm{Bb}}\left(t\right)$, or $x_{\mathrm{bb}}\left(t\right)$ depending on that individual’s genotype; 1 is a column vector of ones, z encodes the genotype of each individual as −1 for BB, 0 for Bb, and 1 for bb, δ(t) is a column vector that contains the residuals, and α(t) is the regression slope capturing the average effect of an allelic substitution, which will depend on the frequency of the genotypes in the population.

Under random mating, α(t) can also be expressed in terms of the additive value a(t), the dominance value d(t), and the allele frequencies (3, Ch. 4] as follows:

[8] $$\begin{array}{cc} \alpha (t) & =a(t)+d(t)(q-p)\\ & =s\left(t\right)\gamma -\left(q-p\right)s^{\left(1\right)}\left(t\right)\frac{\gamma^{2}}{2}+..., \end{array}$$

where we substituted with Eqs. **5** and **6**. Note that only even-order terms are scaled by the allele frequency difference (q−p).

This formulation indicates that α(t) can be expressed as a linear combination of the functions $s\left(t\right),s^{\left(1\right)}\left(t\right),\ldots$, each determined by the underlying developmental dynamics encoded in f. In particular, Eq. **3** shows that s(t) changes dynamically over developmental time, and similar dynamic equations govern its higher-order derivatives—see, e.g., ref. 33 for an explicit formulation of the dynamics of $s^{\left(1\right)}\left(t\right)$. It follows that α(t), being composed of such dynamic components, is itself a dynamic quantity that changes over the course of development.

Assuming that γ is small, as expected under the classical infinitesimal model (3, Ch. 5], we can retain only the leading term in the Taylor expansion. Under this approximation, Eq. **8** simplifies to

[9] $$\begin{array}{c} \alpha (t)\approx s(t)\;\gamma . \end{array}$$

This approximation shows that when allelic effects are small, the average effect α(t) is directly proportional to the sensitivity s(t), and therefore follows the same developmental dynamics.

To illustrate this, Fig. 1*C* compares the average effect α(t) estimated via the classical linear regression given in Eq. **7** at different developmental times t, alongside the quantity s(t)γ, with sensitivity calculated directly from the developmental function f. In this example, the developmental system is a two-gene toggle switch network (*Materials and Methods*), commonly found in cell differentiation (34).

Fig. 1*C* shows that in this two-gene network, α(t) and s(t)γ converge for small γ, as expected by Eq. **9**—even though they are derived from entirely independent approaches: the developmental approach, which integrates developmental dynamics over time (i.e., s(t)), and the statistical approach, which estimates α(t) through linear regression at the population level. While this single-locus example is simple, the analytical derivations extend to more complex systems, as we show in later sections, provided development can be represented as a dynamical system and perturbations remain small.

The relationship between α(t) and s(t) bridges quantitative genetics to a mechanistic view of development for small perturbations. We showed that the average effect behaves as a dynamical system and is proportional to the sensitivity for small perturbations. As mentioned above, this link is central because average effects are a widely used empirical means of linking genetic and phenotypic variation, and because their variance is a key predictor of short-term evolutionary potential. In the next sections, we use this link to i) improve estimates of average effects and ii) examine the relationship between phenotypic (co)variance generated by genetic and environmental sources.

### Estimation of Average Effects from Developmental Trajectories.

Accurate estimation of average genetic effects is central to fields ranging from agriculture to personalized medicine and evolutionary biology. For instance, increasing the accuracy of average effects is critical for refining polygenic scores and improving the prediction of complex phenotypes from molecular genetic data (6). Here, we demonstrate that representing development as a dynamical system enables more precise estimation of these effects by leveraging information from developmental trajectories. This framework is broadly applicable to systems where time-series phenotypic data can be obtained, a capability that is becoming increasingly common for many traits of commercial and clinical interest (1, 35).

Building on the previous section, we use the dynamical nature of the average effect, α(t), to improve its estimation during development. While multiple sources of error can impact these estimates, we show below that many of these can be partially mitigated by leveraging information across the full developmental time series.

As described earlier in the single-locus case, α(t) can be estimated using linear regression. To extend this to multiple loci, we denote by $\alpha_{i}\left(t\right)$ the average effect of an allelic substitution at the i-th locus out of N polymorphic loci. We can write a multiple-loci extension of Eq. **7** for each developmental time t as

[10] $$\begin{array}{c} y\left(t\right)=\mu \left(t\right)\;1+Z\;\alpha \left(t\right)+\epsilon \left(t\right),\;\epsilon \left(t\right)∼N(0,\;\sigma_{\epsilon }^{2}I), \end{array}$$

where y(t) is the column vector of phenotypic values for the $N_{\mathrm{pob}}$ individuals in the population at developmental time t, μ(t) is the baseline phenotype when all genetic predictors are zero, and $Z\in R^{M\times N}$ is the design matrix whose m-th row encodes the genotype of the m-th individual across loci, using values −1, 0, or 1 to encode the genotypes. The column vector $\alpha \left(t\right)=(\alpha_{1}\left(t\right),\cdots ,\alpha_{N}\left(t\right){)}^{T}$ contains the average effects for each of the N loci, and the column vector ϵ(t) contains the residuals, assumed to follow a multivariate normal distribution with independent and identically distributed components of variance $\sigma_{\epsilon }^{2}$. Although additional covariates (e.g., relatedness or environmental factors) are often included in such regressions, here we consider the simplest case: Individuals are unrelated, there is no linkage, and no other covariates are included.

The residual term ϵ(t) captures various sources of variability that hinder the accurate estimation of average effects, as mentioned above. These include deviations arising from unmeasured genetic and environmental factors, as well as measurement error. It also is assumed to include variation generated by nonlinear gene action, such as dominance and epistasis (36)—but note that modeling these effects as noise has been subject to criticism (37, 38).

In addition, ϵ(t) includes contributions from developmental noise, random fluctuations during development that cause phenotypic variation even among individuals with identical genotypes and environments. This noise results from the intrinsic stochasticity of cellular and molecular processes, and while we model it here as uncorrelated Gaussian noise for simplicity, in reality it accumulates over time and introduces temporal correlations.

The regression given in Eq. **10** can be used to estimate the average effects at each developmental time point. We refer to these as the static estimates of the average effect of the i-th locus, denoted $\alpha_{i}^{s}\left(t\right)$. Here, we seek to improve these estimates by leveraging the finding from the previous section that average effects change over developmental time according to a dynamical process. In particular, this dynamic behavior implies a form of memory: The value of $\alpha_{i}\left(t\right)$ depends on its past values (i.e., $\alpha_{i}\left(t-1\right)$, $\alpha_{i}\left(t-2\right)$ and so on), where t indexes discrete time steps of size h=1 for simplicity. This temporal structure can be exploited to obtain more accurate estimates.

To demonstrate this, we model the average effect as a random walk with stochastic increments η(t) having variance $Q_{t}$:

$$\alpha_{i}\left(t\right)=\alpha_{i}\left(t-1\right)+\eta \left(t\right),\;\eta \left(t\right)∼N\left(0,Q_{t}\right),$$

and treat the static estimates $\alpha_{i}^{s}\left(t\right)$ as noisy observations of the true values. We use a Kalman filter (39, 40) to combine these observations with the model, yielding improved estimates $\alpha_{i}^{d}\left(t\right)$. Through its recursive updating mechanism, the Kalman filter integrates information from the entire time series up to time t, allowing each estimate $\alpha_{i}^{d}\left(t\right)$ to reflect the full history of observations. Implementation details of the filter are provided in *SI Appendix*, Text.

We compare the estimation errors of the dynamic and static average effects using the two-gene toggle switch network model described above (*Materials and Methods*). To do this, we simulated a population and generated time series developmental data for each individual, which was then used to compute both estimates and their associated errors.

Estimation errors were assessed across different population sizes and levels of developmental and measurement noise. Developmental noise was introduced as zero-mean Gaussian perturbations with varying variance applied to the states (i.e., gene expression levels) throughout development (*Materials and Methods*). Measurement noise was modeled by adding zero-mean Gaussian noise with varying variance directly to the observed data. Relative errors were computed using analytically derived true average effects based on the sensitivity definition and Eq. **9**. In all simulations, each developmental parameter was determined by 10 underlying loci.

**Fig. 2.**
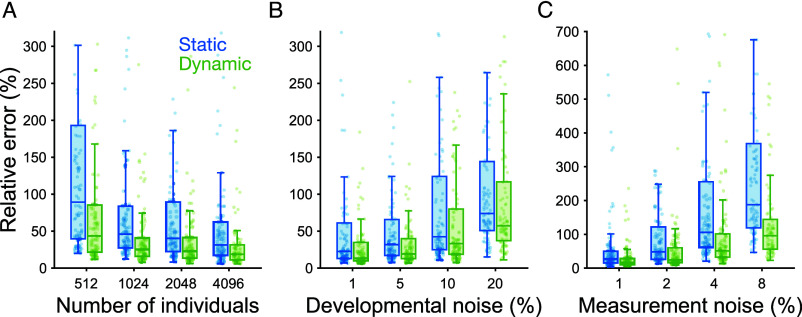
Relative error of dynamic and static estimators of average effects throughout development. Errors were computed as $\left||\alpha_{i}\left(t\right)-s\left(t\right)\gamma_{i}\right|\left|/\left||s\left(t\right)\gamma_{i}\right|\right|$ across all developmental time points, and under varying conditions: population size; measurement noise (introduced as Gaussian noise to the measurements with mean zero and SD 1% to 8% of trait value); and developmental noise (introduced as Gaussian perturbations to states at each time point in development, with mean zero and SD 1% to 20% of trait value). For each parameter combination, 100 replicates were simulated. (*A*) Error versus number of individuals, with both noise sources fixed at 1% to reduce error from other sources. (*B*) Error versus developmental noise, with 4,096 individuals and measurement noise fixed at 1%. (*C*) Error versus of measurement noise, with 4,096 individuals and developmental noise fixed at 1%. Note that noise levels are expressed relative to the trait value, while relative errors are computed with respect to the much smaller average effects.

Fig. 2 shows the relative errors of the dynamic and static estimates of the average effects. The dynamic estimate consistently exhibits lower error than the static estimate because it leverages information from past time points in the developmental trajectory. The improvement is larger when fewer organisms are sampled, and when measurement or developmental noise are higher. However, errors introduced by developmental noise (Fig. 2*B*) are more challenging to correct, as they result in correlated errors in the time series, as explained above.

### Alignment of Phenotypic Variation.

Significant empirical evidence suggests that in many systems, phenotypic (co)variance matrices from different sources of variation, such as genetic and environmental, can be approximately proportional (25, 27, 28). Furthermore, in some lineages, these patterns of standing variation have been found to align with the directions of long-term evolutionary divergence (29, 30). Understanding the mechanistic origins of these statistical patterns is essential for explaining the distribution of phenotypic diversity and the role of plasticity in evolution (12), and carries critical implications for evolutionary prediction (2, 13).

In this section, we build on the link between sensitivity vectors and average effects to derive explicit expressions for phenotypic (co)variance matrices arising from different sources of variation, including genetic variation affecting different developmental processes, environmental fluctuations, and developmental noise. These expressions provide a unified framework for understanding the mechanistic basis of the proportionality and alignment sometimes observed in the literature. While this section focuses on the relationship between (co)variance matrices arising from genetic and environmental variation, the connection to long-term evolutionary diversification is examined in *Discussion*.

We begin by expanding our notation to include multiple traits. We define the column vector $\alpha_{i}\left(t\right)=(\alpha_{i,1}\left(t\right),$$\alpha_{i,2}\left(t\right),\cdots ,\alpha_{i,n}{\left(t\right))}^{T}$ that contains the average effects of the i-th locus for each of the n phenotypic traits of interest, at developmental time t. As before, we assume that each locus i∈{1,⋯,N} affects a single developmental parameter. Let w:{1,⋯,N}→{1,⋯,p} be the function mapping each locus i to the parameter it affects w(i), and write $w_{i}:=w\left(i\right)$; the effect of locus i on $\lambda_{w_{i}}$ has magnitude $\gamma_{i}$. Since this amount is shared across all traits, we can generalize Eq. **9** to the multivariate case as

[11] $$\begin{array}{c} \alpha_{i}\left(t\right)=\gamma_{i}\;s_{w_{i}}\left(t\right), \end{array}$$

which relates the average effects over all relevant traits with the sensitivity vector $s_{w_{i}}\left(t\right)$.

We now express the classical (co)variance matrices of quantitative genetics in terms of the sensitivity vectors. The additive genetic (co)variance matrix G is the variance due to the average effects of alleles. The G-matrix is of special importance in evolutionary quantitative genetics because it is associated to the response to directional selection, evolution under drift, and the evolvability of a population (2, 5).

The additive genetic variance and covariance contributed by the i-th locus in a population at developmental time t, denoted by $G_{i}\left(t\right)$, depends on the average effects of that locus on the traits and on the allele frequencies $p_{i}$ and $q_{i}$ for that locus. Assuming Hardy–Weinberg equilibrium, we have that (41):

$$\begin{array}{c} G_{i}\left(t\right)=2p_{i}q_{i}\;\alpha_{i}\left(t\right)\alpha_{i}^{T}\left(t\right). \end{array}$$

Under the assumption that each locus contributes only a small amount to the developmental parameters, we can use Eq. **11** to approximate

[12] $$\begin{array}{cc} G_{i}\left(t\right) & =2p_{i}q_{i}\;\left(\gamma_{i}\;s_{w_{i}}\left(t\right)\right){\left(\gamma_{i}\;s_{w_{i}}\left(t\right)\right)}^{T}\\ {}[1mm] & =2p_{i}q_{i}\;\gamma_{i}^{2}\;s_{w_{i}}\left(t\right)s_{w_{i}}^{T}\left(t\right)\\ {}[1mm] & =\sigma_{i}^{2}\;s_{w_{i}}\left(t\right)s_{w_{i}}^{T}\left(t\right), \end{array}$$

where we have expressed the contribution of additive genetic variation of a single locus as a function of the sensitivity vector. Here, $\sigma_{i}^{2}=2p_{i}q_{i}\;\gamma_{i}^{2}$ represents the variance in the developmental parameter associated with locus i and is thus a property of the population. Finally, under the assumption of small perturbations—consistent with the classical assumptions of the infinitesimal model—the full G-matrix is given by the sum of contributions from all polymorphic loci, i.e., $G\left(t\right)=\sum_{i}G_{i}\left(t\right)$. The notation emphasizes that the G-matrix is a function of developmental time (42).

The formalism of sensitivity vectors also allows us to represent the phenotypic variation generated by environmental factors in a manner analogous to the genetic contributions discussed above. To illustrate this, consider an environmental variable j (e.g., temperature) that perturbs the developmental system. Let v be the function that maps the environmental variable j to the affected developmental parameter $v_{j}$, and $\sigma_{j}^{2}$ denote the variance in the developmental parameter generated by the perturbation. For simplicity, we assume the environmental factor influences only a single parameter, though this approach readily extends—under the assumption of small perturbations—to multiple parameters contributing independently to the overall variance. To a first-order approximation, the variance and covariance resulting from this environmental perturbation can be expressed as

$$\begin{array}{c} E_{j}\left(t\right)=\sigma_{j}^{2}\;s_{v_{j}}\left(t\right)\;s_{v_{j}}^{T}\left(t\right). \end{array}$$

Summing across all perturbed environmental variables gives the overall environmental matrix, $E\left(t\right)=\sum_{j}E_{j}\left(t\right)$. Finally, and again under the assumption of small perturbations, the total phenotypic (co)variance matrix can be approximated as P(t)=G(t)+E(t).

Expressing the (co)variance matrices as sums over sensitivity vectors highlights how their structure depends on both the orientation of these vectors—set by the developmental function f—and the magnitude of variation in the underlying factors, which reflects population-level properties.

This provides a framework for analyzing and interpreting the relationship between covariance matrices generated by different sources of variation, a central topic in the study of biological variation (25–29). It has been suggested, for example, that the phenotypic (P) and genetic (G) covariance matrices are often approximately proportional. This observation, sometimes referred to as Cheverud’s conjecture (25), has received empirical support in a variety of systems (43, 44). However, the conditions under which such proportionality should be expected remain unclear (27). The framework we present here helps to clarify this issue by showing that proportionality between (co)variance matrices depends on the joint satisfaction of two distinct conditions: one developmental and one population-level.

The developmental condition requires an alignment between the sensitivity vectors corresponding to genetic and environmental perturbations. This alignment condition arises solely from the structure of the developmental function f, and prior theoretical work suggests that it is likely to be satisfied under a wide range of perturbations (13, 45, 46). The population condition, on the other hand, requires that there is sufficient variation in the underlying perturbations associated to the aligned sensitivity vectors, so that this direction dominates the overall structure of the (co)variance matrices.

**Fig. 3.**
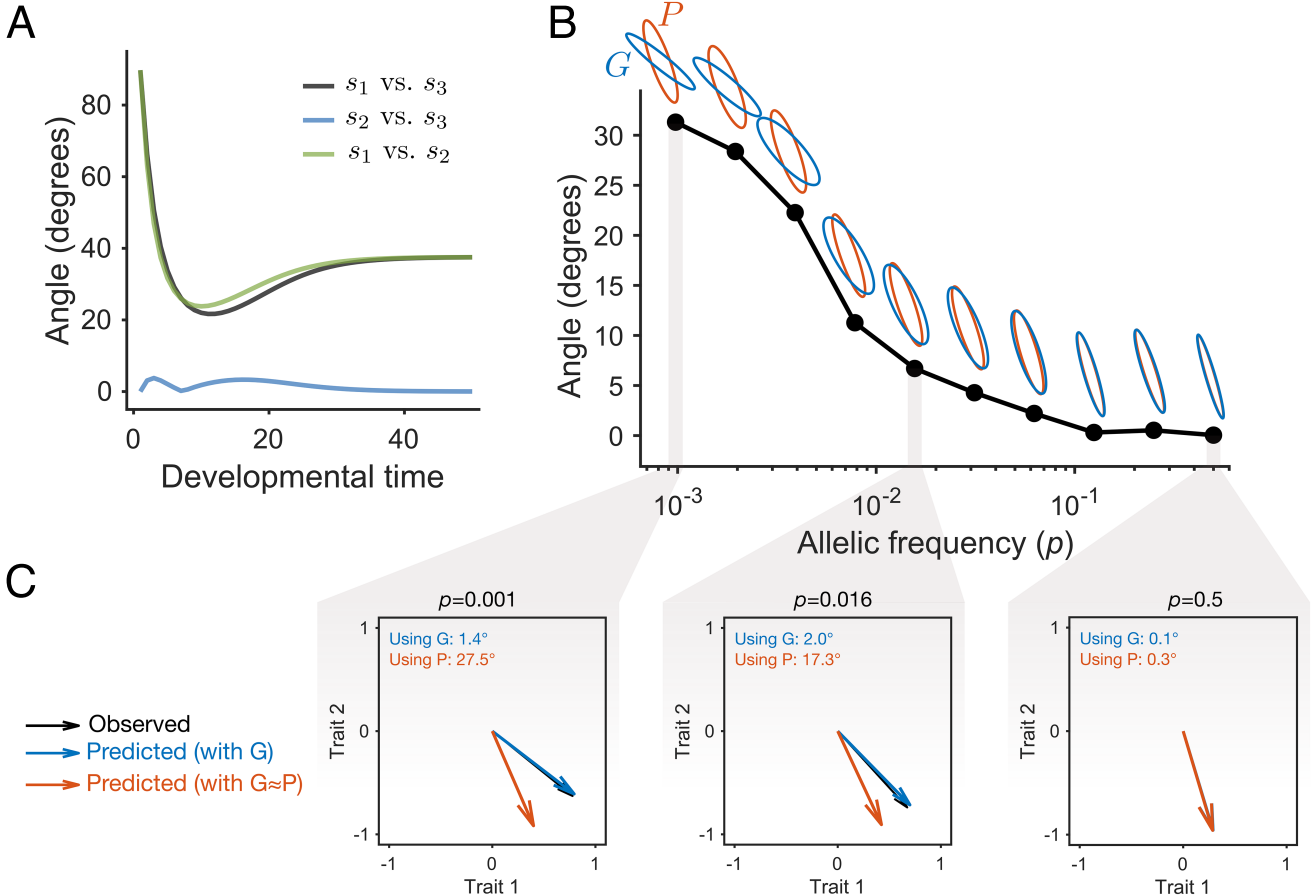
Proportionality between covariance matrices depends on sensitivity vectors and underlying variation, and affects evolutionary predictions. (*A*) Angles between the sensitivity vectors for the developmental parameters $\lambda_{1}$, $\lambda_{2}$, and $\lambda_{3}$, calculated by integrating Eq. **3**. Notably, the sensitivity vectors for $\lambda_{2}$ and $\lambda_{3}$ are largely aligned, implying that perturbations in either parameter yield similar phenotypic variation. (*B*) Angle between $G_{\max}$ and $P_{\max}$ at developmental time 50—a measure of proportionality between G and P—against the minor allele frequency (p) associated with variation in $\lambda_{2}$, while environmental variation in $\lambda_{3}$ is held constant. As p approaches 0.5, population-level variation in $\lambda_{2}$ increases, enhancing G-P proportionality. For each point, G and P are shown normalized by their norms for comparison. (*C*) We simulated one generation of evolution across allele frequencies and compared the observed change in trait space with predictions from the multivariate breeder’s equation (vectors shown normalized). The angle between predicted and observed changes is small when using G, whereas predictions based on P are misaligned at low allele frequencies.

To illustrate these points, we revisit the gene regulatory network example introduced earlier, now extended to include an additional developmental parameter $\lambda_{3}$ that is influenced by an environmental variable (*Materials and Methods*). In Fig. 3*A*, we show the angles between sensitivity vectors for each parameter through development. The parameters $\lambda_{1}$ and $\lambda_{2}$ are genetically determined—each influenced by ten loci—while $\lambda_{3}$ is environmentally determined. The figure indicates that the sensitivity vectors corresponding to $\lambda_{2}$ and $\lambda_{3}$, but not $\lambda_{1}$, are closely aligned (i.e., form a small angle), reflecting similar effects on the phenotype throughout development. This alignment indicates that $\lambda_{2}$ and $\lambda_{3}$ satisfy the developmental condition.

However, as noted earlier, this developmental condition alone is not sufficient for proportionality between the genetic and phenotypic covariance matrices. For proportionality to emerge, the population condition must also be met—that is, there must be sufficient variation in both $\lambda_{2}$ and $\lambda_{3}$. Only when both the direction of effect (developmental condition) and the magnitude of variation (population condition) are met can the genetic (G) and environmental (E) contributions align, resulting in proportionality between the genetic (G) and phenotypic (P) covariance matrices.

To illustrate this, Fig. 3*B* shows the proportionality between G and P as a function of standing genetic variation in the loci affecting the parameter $\lambda_{2}$, while keeping environmental variation in $\lambda_{3}$ constant. The matrices are computed using phenotypes at the final developmental time point (i.e., t=50 in Fig. 3*A*, see *SI Appendix*, Fig. S1 for results at an earlier developmental stage). Since the system has only two phenotypic states, we quantify proportionality directly using the angle between $G_{\max}$ and $P_{\max}$. For systems with more traits, proportionality can be better assessed by evaluating the similarity in variance distribution across orthogonal axes (26, 28).

Within the population, variation in the loci underlying $\lambda_{2}$ is maximized when the minor allele frequencies approaches 0.5 and decreases as the frequency approaches 0. As expected, the alignment between G and P increases with the amount of genetic variation; when the minor allele frequency nears 0.5, there is almost complete proportionality. Thus, even though development may inherently favor alignment between genetic and environmental effects, sufficient variation in the underlying factors is essential to reveal this proportionality at the population level.

This alignment between G and P has direct implications for evolutionary prediction. As mentioned above, P is often used as a proxy for G when the latter cannot be estimated (25, 47, 48), but misalignment can lead to inaccurate predictions. To illustrate this, we simulated a single generation of evolution in our gene network model, selecting individuals closest to an arbitrary optimum phenotype of 4 for the two traits, at minor allele frequencies (p) of 0.001, 0.016, and 0.5. Selected individuals were randomly paired, and offspring were generated via recombination. We then predicted the change in population mean using the multivariate breeder’s equation (5), which relates the evolutionary response to selection through the additive genetic covariance matrix G and the selection gradient. Predictions were made using either the true G or P as a proxy. Fig. 3*C* shows that predictions based on G consistently show a small angle with the observed change, whereas those based on P were poor at low allele frequencies due to misalignment. This demonstrates that whether the developmental and population-level conditions are met directly affects the accuracy of evolutionary predictions.

While the gene regulatory network model used here is a simplified representation of development, the alignment of sensitivity vectors is expected to arise more generally—even in more complex developmental systems. Indeed, general theoretical conditions for such alignment have been established in previous work (13; see also refs. 46 and 49 and *Discussion*), showing that it arises when perturbations influence similar dynamical aspects of development.

To show this, we used a model of tooth development (50; see *Materials and Methods*). Although this model is considerably more complex than the previously discussed gene regulatory network, it is built on the same core principle: Development is modeled as a dynamical system, with developmental parameters affecting its behavior over time. This model was previously used to demonstrate that the breeder’s equation (5) can fail to predict short-term evolution for nonlinear genotype-phenotype maps (21); here, we use it to show that distinct perturbations can induce aligned phenotypic effects also in developmental systems consisting of several components and processes.

**Fig. 4.**
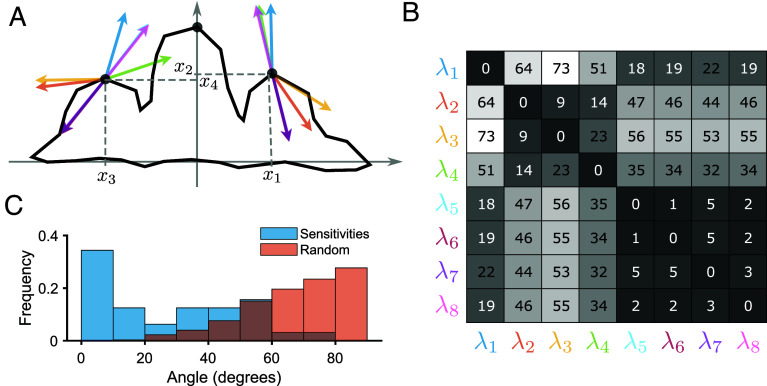
Alignment in the tooth model. (*A*) Outline of a tooth generated by the model using the reference parameter values. The four traits measured $\left(x_{1},x_{2},x_{3},x_{4}\right)$ correspond to the positions of two landmarks at the anterior and posterior cusps. The arrows represent the sensitivity vectors for eight parameters, scaled by their norm for ease of comparison. (*B*) Pairwise angles between sensitivity vectors, with colors corresponding to those of the arrows in (*A*); there are clusters of parameters with small angles. (*C*) Distribution of angles between sensitivity vectors, compared with the distribution of random angles in 4-dimensional space (equal to the number of traits measured). The sensitivity angles are significantly closer to zero than the random angles.

The tooth model explicitly simulates a sheet of epithelial cells that divide and fold to form tooth morphology (Fig. 4*A*), mirroring real developmental processes. It integrates the known gene regulatory network—including an activator, inhibitor, and secondary signal—alongside the mechanical interactions and cell behaviors necessary for tooth formation.

We perturbed eight developmental parameters in the tooth model and estimated the resulting sensitivity vectors via regression, as described in the previous section. The phenotypic effects of these perturbations were quantified on four traits—the (x,y) coordinates of two landmarks at the anterior and posterior cusps. Fig. 4*A* shows the morphology of the reference tooth, along with the 8 calculated sensitivity vectors at a fixed developmental time corresponding to 8,000 iterations of the simulation.

Fig. 4*B* shows clusters of sensitivity vectors with small intervector angles, indicating a notable degree of alignment. Moreover, Fig. 4*C* demonstrates that these angles are significantly smaller than those between random vectors in the 4-dimensional trait space.

One cluster comprises the sensitivities associated with parameters 5, 6, 7, and 8, all of which contribute to the reaction–diffusion mechanism of the developmental system (50). These parameters correspond, respectively, to the autoactivation of the activator molecule, the inhibition of the activator by the inhibitor, the diffusion rate of the activator and the diffusion rate of the inhibitor. Perturbations in any of these parameters yield similar phenotypic effects, as expected from their close involvement in the same developmental process.

In contrast, parameters 2, 3, and 4—which correspond to the secretion rate of the secondary signaling molecule, the mechanical resistance of the mesenchyme, and the protein degradation rate, respectively—also show a high degree of alignment, even though they do not obviously influence the same aspect of development. This unexpected alignment could be explained by properties of the Jacobian matrix A in Eq. **3** (see *Discussion* and ref. 13). Finally, parameter 1, the epithelial growth rate, appears to affect the phenotype in a distinct manner to the other parameters.

Overall, these findings suggest that even complex developmental systems often tend to align the phenotypic effects of diverse perturbations (13, 49, 51). Consequently, multiple sensitivity vectors are likely to satisfy the developmental alignment condition described above. This, in turn, increases the likelihood of proportionality among phenotypic (co)variance matrices (Fig. 3*B*), as many perturbations can induce sufficient variation in the relevant developmental components for the population condition to be met.

## Discussion

In this work, we establish a formal bridge between developmental dynamics and the statistical framework of quantitative genetics. Specifically, by representing development as a dynamical system (Eq. **1**; see refs. 8, 9, and 23) and applying the formalism of sensitivity vectors (13), we derive general expressions linking developmental processes to key statistical quantities. This framework is not tied to any particular mechanistic model but instead captures broad features of development that can inform statistical analyses. Through this approach, we show how developmental insights can enhance both the estimation of quantitative genetic parameters and the interpretation of patterns of standing variation and evolutionary change.

The sensitivity framework used here differs in important ways from other approaches that link development to population-level variation (52, 53, 54; see also refs. 14, 15, and 18) Crucially, the sensitivities in our framework are explicitly dynamic: They are functions of developmental time. This contrasts models that express phenotype as a nonlinear function of underlying factors evaluated at a fixed developmental stage (e.g., the adult form; 52, 53). This dynamic perspective enables two key results: improved estimation of statistical parameters when using developmental data and a mechanistic analysis of the alignment between sources of variation and phenotypic covariation, as discussed below.

Our explicitly dynamic approach expresses the average effects of alleles—classically defined in quantitative genetics as the mean phenotypic deviation of an individual carrying a given allele relative to the population mean—in terms of sensitivity vectors (Eq. **9**). This representation enables the use of recursive models to track how these effects change over developmental time. By leveraging the full time series of developmental data, this framework improves the estimation of average effects, particularly in contexts with few individuals or high measurement noise. This can enhance estimates of key quantities such as polygenic scores, offering a concrete example of how explicitly incorporating developmental dynamics and complete time series data can sharpen inferences about the genotype-phenotype map and its evolutionary implications (55). Time-series data of this kind can be obtained by high-throughput phenotyping through developmental time (1, 35), underscoring the need for technologies such as automated, nondestructive tracking of traits–like those developed for root-system architecture or biomass accumulation (56).

Beyond improving estimation, considering complete developmental trajectories is essential because evolutionary processes can act at different developmental stages: Selection can vary across development, and additive genetic variance often changes through ontogeny (55, 57, 58). These considerations link our framework to longstanding questions about the evolution of developmental trajectories, traditionally addressed with the function-valued trait framework (55, 57). The two approaches are complementary and compatible: Both aim to understand evolutionary dynamics by considering entire trajectories rather than only end-point phenotypes. They differ mainly in representation. The function-valued trait framework models trajectories statistically as combinations of basis functions (e.g., polynomials or splines), which are powerful for quantifying variation and selection over time but are not tied to explicit development. In contrast, our approach defines trajectories as solutions of a developmental dynamical system, as is common in developmental biology (23), allowing the use of tools such as sensitivity analysis and Jacobian structure to connect statistical patterns of variation directly to underlying developmental processes. As we discuss next, this is what forms the basis for the analyses of alignment of perturbation effects.

Specifically, we identify two sufficient conditions for proportionality to arise between covariance matrices originating from different sources (e.g., genetic or environmental). First, the developmental condition requires that small perturbations induce shifts in phenotype along similar directions in trait space, which in our framework corresponds to sensitivity vectors that point in similar directions. The developmental condition is expected to be fulfilled when perturbations affect the same dynamical features of the system. In this context, the developmental dynamics determined by networks of interactions among genes, cells, and tissues, act as a “funnel” that channels diverse underlying factors into constrained phenotypic outcomes. As a result of this funneling, many distinct perturbations result in similar phenotypic effects (13, 45, 59, 60).

We can formalize this intuitive notion of “funneling” of perturbations within our framework by examining the developmental function f, and the elements b and A in Eq. **1** (13, see also refs. 46 and 49). For sensitivity vectors to be aligned (i.e., fulfillment of developmental condition), it is sufficient that perturbations have proportional vectors b, indicating that the parameters affect f in similar ways. This scenario can be facilitated by common developmental architectures, such as the bow-tie structure (61), where many inputs converge on a small set of intermediate regulators before diverging again—for example, in the shavenbaby gene network in *Drosophila* (62). Alignment of sensitivity vectors is also promoted when the inverse Jacobian $A^{-1}$ has columns that are nearly linear combinations of each other, or equivalently, when one or a few eigenvalues dominate its spectrum. In this case, the system is effectively organized around a small number of dominant modes, causing a broad range of perturbations to converge on these modes and produce similar phenotypic effects (13, 46).

Experimental perturbations of development is the most appropriate approach to evaluate whether developmental dynamics act as a funnel and the developmental condition is fulfilled. Empirical evidence consistent with such funneling has been obtained, particularly in microorganisms where perturbations can be systematically applied. For example, experiments manipulating both genetic and environmental variation in *Escherichia coli* have shown that transcriptomic responses are strongly aligned along a conserved mode of variation (45, 46, 63). In the context of our framework, such results can be interpreted as direct evidence that distinct perturbations induce aligned phenotypic shifts, corresponding to sensitivity vectors that point in similar directions and thus satisfy the developmental condition. More generally, this approach involves applying multiple, distinct perturbations to the same developmental system and assessing whether the resulting phenotypic shifts are aligned in trait space.

The fulfillment of the developmental condition alone, however, is not enough to guarantee proportionality among (co)variance matrices. As shown here, these matrices depend not only on developmental sensitivity but also on the distribution of underlying sources of variation. Therefore, a second requirement which we call the population condition must also be satisfied. This requires that there is sufficient variation in those specific underlying factors whose effects are aligned according to the developmental condition. When such factors account for a substantial proportion of total phenotypic variance, their aligned effects dominate the phenotypic covariance structure. This allows the alignment to be empirically observed when comparing covariance matrices estimated from standing variation.

The role of the population condition is illustrated in Fig. 3, where we examine the proportionality between the G and P matrices. In this example, the developmental condition is always met (Fig. 3*A*), so any variation in the proportionality between G and P arises solely from the population condition—specifically, differences in allelic frequencies (Fig. 3*B*). This example highlights that inferring underlying mechanisms from observed statistical patterns can be misleading, as population structure and distributions may distort or obscure the developmental signal (64–66).

The same reasoning used to interpret the proportionality between G and P extends to other phenotypic (co)variance matrices, including those shaped by novel mutations or developmental noise. Such proportionality among phenotypic (co)variation of different origins has been increasingly documented across a wide range of biological systems (26, 28–30). For example, analyses of *Drosophila melanogaster* wings using landmark-based approaches show that phenotypic variation from genetic, environmental, and developmental sources is aligned along similar directions in morphospace (26, 28, 67). These directions appear to be shared across species (26, 28), suggesting that some features of this developmental funneling process are evolutionarily conserved. According to our framework, this cross-scale alignment arises when perturbations at different levels influence shared dynamical features of development (satisfying the developmental condition) and do so with sufficient variance to leave a detectable statistical signature (satisfying the population condition). Deviations from this alignment are therefore expected when either condition is not met, and identifying which condition fails in specific empirical cases offers a promising direction for future research.

Provided that the developmental funnel is preserved over time, as expected when the underlying dynamical structure is maintained (13), the framework presented here can help to explain two fundamental patterns in evolution.

First, recent studies have reported a persistent alignment between the major axes of additive genetic variation within populations (G-matrices) and long-term evolutionary divergence—that is, evolutionary change in population or species mean phenotypes (26, 29). This alignment has proven difficult to explain (30). However, if sensitivity vectors remain conserved, genetic perturbations will tend to accumulate along the directions defined by those vectors, unless consistently selected against. These same conserved directions will also determine the structure of standing genetic variation, as shown in Eq. **12**, provided the population condition is also met. Thus, when the dynamical properties of the developmental system are stable, and the population condition is fulfilled, an alignment between standing genetic variation and long-term evolutionary divergence is expected. Conversely, evolution of the developmental mechanisms itself can allow exploration of novel areas of morphospace because the variational properties change (68). It remains an open question when and why the underlying dynamical structure of development remains conserved over long evolutionary timescales (26, 29, 30).

A second, related observation is the long-term stability of certain aspects of genetic covariance matrices (G-matrices) observed in some empirical datasets—e.g., shape variation in dipteran wings (26, 28, 69). In our framework, this stability requires the conservation of the major axes of variability of the developmental system, along with the fulfillment of the population condition. Violating either of these conditions should therefore disrupt this stability. For instance, G-matrices can change rapidly when population properties shift—such as during bottlenecks that alter the distribution of genetic variation (70). Likewise, changes in the major axes of variability of the developmental system can also lead to changes in the G-matrix, as they are associated with changes in the directions of the sensitivity vectors. This is expected to occur when populations traverse complex, nonlinear regions of the genotype-phenotype map (38, 52). Consequently, when changes in genetic (co)variances are observed, an important empirical question is whether they reflect changes in development, evidenced by altered sensitivity vectors, or changes in population properties such as allele-frequency shifts from demographic events (e.g., bottlenecks).

In summary, the work presented here provides a conceptual and analytical foundation for integrating evolutionary developmental biology with microevolutionary theory (71, 72)—an essential step toward a more complete understanding of how development shapes heritable variation, and how that variation interacts with natural selection and other population-level processes.

## Materials and Methods

### Gene Regulatory Network Model.

We used the bistable switch gene regulatory network as a simplified model of development, following ref. 34. The system is defined by a pair of coupled nonlinear ordinary differential equations:

$$\begin{array}{cc} {\dot{x}}_{1} & =\frac{2+\lambda_{1}}{1+{\left(\frac{x_{2}}{2}\right)}^{2}}-0.4\;x_{1},\\ {\dot{x}}_{2} & =\frac{2+\lambda_{2}}{1+{\left(\frac{x_{1}}{3}\right)}^{2}}-0.4\;x_{2}, \end{array}$$

where $x_{1}$ and $x_{2}$ denote the expression levels of genes 1 and 2, respectively. $\lambda_{1}$ and $\lambda_{2}$ are developmental parameters that modulate the regulatory input each gene receives. Each parameter is determined by the sum of contributions from multiple genetic loci, with each locus contributing additively and amount $\gamma_{i}$. Unless otherwise specified, we simulated 10 loci for each developmental parameter, and the values $\gamma_{i}$ were randomly sampled from a Gaussian distribution of mean 0 and SD 0.01.

For the simulations presented in Fig. 3, we incorporated an environmentally determined developmental parameter $\lambda_{3}$, modifying the regulation of $x_{2}$ by replacing the denominator term ${\left(\frac{x_{1}}{3}\right)}^{2}$ with ${\left(\frac{x_{1}}{3+\lambda_{3}}\right)}^{2}$ (73).

For the evolutionary simulations in Fig. 3*C*, we generated populations of 5,000 individuals with genotypes at 10 loci per genetically determined developmental parameter ($\lambda_{1}$ and $\lambda_{2}$). The minor allele frequency for loci underlying $\lambda_{1}$ was fixed at 0.5, whereas that for $\lambda_{2}$ varied (P=0.001,0.016,0.5) to test different levels of alignment. In all cases, the contribution of each locus to its corresponding developmental parameter ($\gamma_{i}$) was drawn from a Gaussian distribution with mean 0 and SD $10^{-4}$. The environmentally determined parameter $\lambda_{3}$ was sampled independently for each individual from a Gaussian distribution with mean 0 and SD $1.5\times 10^{-3}$. Phenotypes were obtained by simulating the gene network until developmental time 50. This initial set of individuals constituted the parental generation, which was then subject to selection: The 50% closest to an arbitrary optimum of (4,4) were chosen as parents and randomly paired. From each pair, four offspring were generated by recombination, assuming no linkage. Developmental parameters for each offspring were calculated from their recombinant genotypes, and phenotypes were obtained as before, with new random values of $\lambda_{3}$. The difference between the parental and offspring means was taken as the realized evolutionary change, which we compared to predictions from the multivariate breeder’s equation.

### Tooth Model.

The tooth model (50) simulates the early stages of tooth formation, beginning with a flat epithelium that gradually develops into a complex three-dimensional structure. It integrates a known gene regulatory network—including an activator, inhibitor, and secondary signal—alongside the mechanical interactions and cell behaviors required for tooth morphogenesis.

The developmental process is governed by a set of parameters that quantify cellular behaviors and molecular interactions. Variations in these parameters alter the dynamics of development and ultimately shape the final three-dimensional distribution of cells. Here, we explore variation in the following parameters: $\lambda_{1}$, epithelial growth rate; $\lambda_{2}$, secretion rate of the secondary signaling molecule; $\lambda_{3}$, mechanical resistance of the mesenchyme; $\lambda_{4}$, protein degradation rate; $\lambda_{5}$, autoactivation of the activator molecule; $\lambda_{6}$, inhibition of the activator by the inhibitor; $\lambda_{7}$, diffusion rate of the activator; and $\lambda_{8}$, diffusion rate of the inhibitor. For each simulated tooth, we quantified developmental traits by measuring the x and y coordinates of two landmarks, as shown in Fig. 4*A*.

Further details of the model are provided in the original publication (50) and in subsequent work using the model (38).

## Supplementary Material

Appendix 01 (PDF)

## Data Availability

Simulation results and code to generate data are deposited in GitHub at https://github.com/lisandromilocco/DevStat-Bridge (74).
