# Supplementary material for: Bridging developmental and statistical approaches to variation and evolution
Source: Proc Natl Acad Sci U S A. 2026 Mar 11;123(11):e2529820123. doi: 10.1073/pnas.2529820123 (PMC12993955; doi:10.1073/pnas.2529820123)
Supplement: Supplementary file 1 — Appendix 01 (PDF) [file pnas.2529820123.sapp.pdf]

Supporting Information for

Bridging developmental and statistical approaches to variation and evolution

Lisandro Milocco and Tobias Uller

Lisandro Milocco.
E-mail: lisandro.milocco@zoologi.su.se

This PDF file includes:

Supporting text
Figs. S1 to S2
SI References

Supporting Information Text

Appendix A: Continuity of Solutions and Validity of Sensitivity Approximations

Here, we provide a justification for the Taylor expansion around a reference trajectory for small perturbations within a finite time window. In our model, developmental trajectories are described by a system of ordinary differential equations (ODEs)

$$\dot{\boldsymbol{x}} = \boldsymbol{f}(t, \boldsymbol{x}, \boldsymbol{\lambda}),$$

where the state variable $\boldsymbol{x}(t) \in \mathbb{R}^n$ represents a vector of developmental quantities (e.g., gene expression or morphogen concentrations), and $\boldsymbol{\lambda} \in \mathbb{R}^p$ represents developmental parameters (e.g., kinetic rates).

We focus on finite developmental intervals $[t_0, t_1]$, corresponding to the finite period of biological development that we are interested in. This finite-time assumption is essential both biologically—because development occurs over bounded timescales—and mathematically—because it ensures that the solution remains well-defined and that the Taylor expansion of the trajectory with respect to parameter perturbations remains valid for sufficiently small perturbations.

1. Continuity with respect to parameters. Let $\boldsymbol{x}(t, \boldsymbol{\lambda}^*)$ be the reference developmental trajectory corresponding to reference parameter values $\boldsymbol{\lambda}^*$ and initial condition $\boldsymbol{x}_0$. The following theorem adapted from *Theorem 3.5* in (1), formalizes the idea that small parameter perturbations yield small deviations in the resulting trajectory. For the complete proof of the theorem, please see (1).

Theorem (Continuity of solutions w.r.t. parameters)**.** *Let $\boldsymbol{f}(t, \boldsymbol{x}, \boldsymbol{\lambda})$ be continuous in $(t, \boldsymbol{x}, \boldsymbol{\lambda})$ and locally Lipschitz in $\boldsymbol{x}$ (uniformly in t and $\boldsymbol{\lambda}$) on $[t_0, t_1] \times D \times \{\|\boldsymbol{\lambda} - \boldsymbol{\lambda}^*\| < c\}$, where $D \subset \mathbb{R}^n$ is open and connected. Let $\boldsymbol{x}(t, \boldsymbol{\lambda}^*)$ be the solution of*

$$\dot{\boldsymbol{x}} = \boldsymbol{f}(t, \boldsymbol{x}, \boldsymbol{\lambda}^*), \qquad \boldsymbol{x}(t_0, \boldsymbol{\lambda}^*) = \boldsymbol{x}_0 \in D,$$

and suppose $\boldsymbol{x}(t, \boldsymbol{\lambda}^*)$ remains in D for all $t \in [t_0, t_1]$. Then, for any $\varepsilon > 0$, there exists $\delta > 0$ such that if $\|\boldsymbol{\lambda} - \boldsymbol{\lambda}^*\| < \delta$, the solution $\boldsymbol{x}(t, \boldsymbol{\lambda})$ of

$$\dot{\boldsymbol{x}} = \boldsymbol{f}(t, \boldsymbol{x}, \boldsymbol{\lambda}), \qquad \boldsymbol{x}(t_0, \boldsymbol{\lambda}) = \boldsymbol{x}_0,$$

satisfies

$$\|\boldsymbol{x}(t, \boldsymbol{\lambda}) - \boldsymbol{x}(t, \boldsymbol{\lambda}^*)\| < \varepsilon, \qquad \forall\, t \in [t_0, t_1].$$

2. Geometric interpretation: a *tube* around the reference trajectory. Because the reference trajectory $\boldsymbol{x}(t, \boldsymbol{\lambda}^*)$ is continuous and bounded on $[t_0, t_1]$ and we assume that $\boldsymbol{f}$ is smooth, the theorem states that we can define a *tube* of radius ε around the reference trajectory:

$$U := \left\{(t, \boldsymbol{x}) \in [t_0, t_1] \times \mathbb{R}^n \;\middle|\; \|\boldsymbol{x}(t, \boldsymbol{\lambda}) - \boldsymbol{x}(t, \boldsymbol{\lambda}^*)\| \le \varepsilon\right\}.$$

This tube, illustrated in Fig. S2, contains all states that stay within a tolerance ε of the reference trajectory. The theorem ensures that there exists $\delta > 0$ such that if $\|\boldsymbol{\lambda} - \boldsymbol{\lambda}^*\| < \delta$, the perturbed trajectory $\boldsymbol{x}(t, \boldsymbol{\lambda})$ remains entirely inside U for $t \in [t_0, t_1]$. This guarantees that, for sufficiently small perturbations, the Taylor expansion of the developmental trajectory in terms of parameter deviations is well defined.

3. Implications. The continuity theorem guarantees that the Taylor approximation around the reference trajectory remains accurate for sufficiently small perturbations and over a finite developmental window $[t_0, t_1]$, which is the regime relevant for studying small genetic or environmental perturbations in development.

Appendix B: Filtering Approach for Improved Estimates of Average Effects

Here, we explain the adaptive Kalman filtering approach used to obtain the dynamic estimates $\alpha_i^d(t)$ for the average effect of the i-th locus, by smoothing the noisy static estimates $\alpha_i^s(t)$ using temporal information from the entire series.

We model the latent process and noisy observations using the following scalar linear Gaussian state-space model:

$$\begin{aligned} \text{State Equation:} \quad & \alpha_i(t) = \alpha_i(t-1) + \eta(t), \\ \text{Observation Equation:} \quad & \alpha_i^s(t) = \alpha_i(t) + \epsilon(t), \end{aligned}$$

where $\alpha_i(t)$ is the true latent state at time t, and $\alpha_i^s(t)$ is the observed static estimate. The process noise $\eta(t) \sim \mathcal{N}(0, Q_t)$ is Gaussian with variance Q_t to be estimated. The observation noise $\epsilon(t) \sim \mathcal{N}(0, R_t)$ is also Gaussian with variance R_t, which is likewise estimated from the data. The Kalman filter also estimates the uncertainty in the latent state, denoted P_t, which represents the variance of $\alpha_i^d(t)$ given the observations up to time t.

Step 1: Estimating Observation Noise Variance. To estimate R_t, we define a local window of length L ending at time t:

$$\tau \in \{\max(1, t-L+1), \dots, t\}.$$

The window length is chosen under the assumption that both R_t and Q_t remain approximately constant over the window, and that the average effect changes roughly linearly during this period. These assumptions allow for stable estimation in a small window while still capturing local dynamics. While L can be selected automatically using criteria such as AIC, we fix $L = 10$ for simplicity (other values yield similar results).

We fit a linear trend to the static estimates $\alpha_i^s(\tau)$ within the window, yielding fitted values $\hat{y}(\tau)$. Assuming the trend captures the underlying signal reasonably well, we treat deviations from it as measurement noise (2). The observation noise variance is then estimated as the residual variance:

$$R_t = \text{Var}(\alpha_i^s(\tau) - \hat{y}(\tau)).$$

Step 2: Estimating Process Noise Variance. We define the process noise variance Q_t as a scaled version of the observation noise:

$$Q_t = \rho \cdot R_t,$$

and search over a grid of ρ values (here logarithmically spaced over $[10^{-2}, 10^2]$) (3). For each candidate ρ, we restart a Kalman filter at the beginning of the window using the optimal values of $\alpha_i^d(t-L)$ and P_{t-L} obtained previously at time $t-L$, and apply it forward to time t, producing dynamic estimates $\alpha_i^{d,(\rho)}(\tau)$ over the window. To select the best ρ, we compute the sum of squared deviations from the fitted trend $\hat{y}(\tau)$:

$$\text{SSE}(\rho) = \sum_{\tau} \left(\alpha_i^{d,(\rho)}(\tau) - \hat{y}(\tau) \right)^2.$$

We then retain the ρ (and corresponding Q_t) that minimizes this quantity.

Step 3: Final Kalman Filter Update. Once ρ_t and Q_t are selected, we perform a single Kalman update at time t, chaining from the previous time step. We define $\alpha_i^d(t-1)$ and P_{t-1} be the filtered mean and variance from the previous step, with values for initialization $P_0 = 1$ and $\alpha_i^d(0) = \alpha_i^s(1)$. We then compute the Kalman update equations (4):

$$\begin{aligned} K_t &= \frac{P_{t-1} + Q_t}{P_{t-1} + Q_t + R_t}, \\ P_t &= (1 - K_t) \cdot (P_{t-1} + Q_t), \\ \alpha_i^d(t) &= \alpha_i^d(t-1) + K_t \cdot (\alpha_i^s(t) - \alpha_i^d(t-1)). \end{aligned}$$

This yields the updated filtered estimate $\alpha_i^d(t)$.

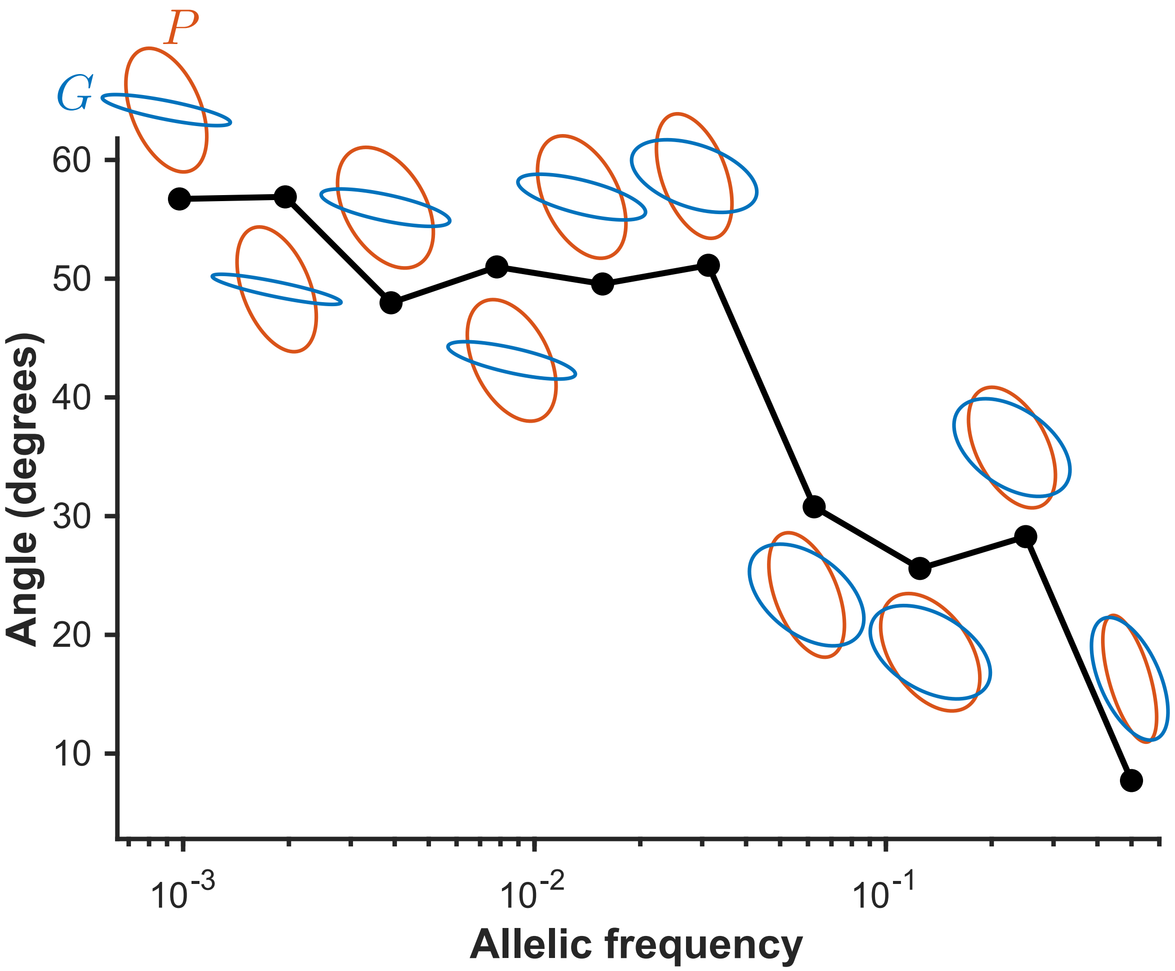

Fig. S1. Proportionality between covariance matrices depends on both sensitivity vectors and the underlying sources of variation. The figure, equivalent to main Fig. 3b but at earlier developmental time $t = 1$, shows the angle between $G_{\max}$ and $P_{\max}$ plotted against the minor allele frequency (p) associated with variation in λ_2, while environmental variation in λ_3 is held constant. As p approaches 0.5, population-level variation in λ_2 increases, leading to stronger G–P proportionality. However, G and P are less aligned here than in Fig. 3b because the sensitivity vectors themselves are less aligned at this earlier developmental stage (see Fig. 3a). This illustrates that the degree of alignment between genetic and phenotypic variation changes throughout development.

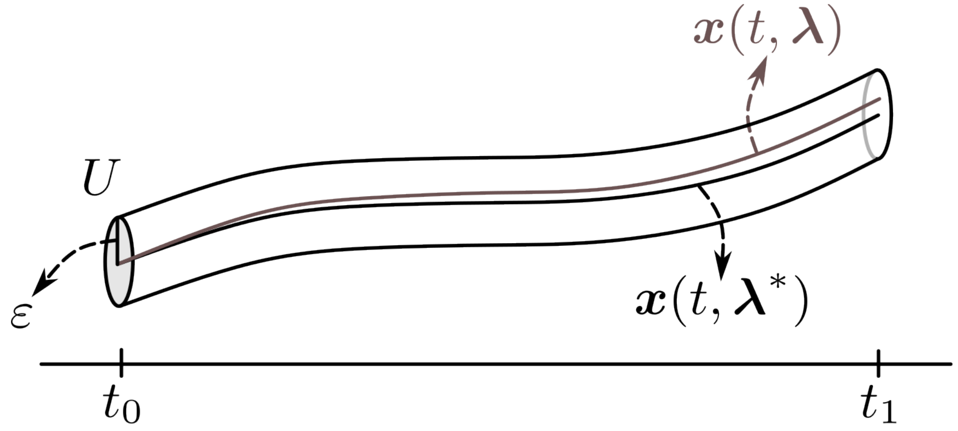

Fig. S2. Illustration of the “tube” U around the reference developmental trajectory $\boldsymbol{x}(t, \boldsymbol{\lambda}^*)$. Each cross-section of the tube (grey region) represents all states within ε of the reference trajectory at a given time. For sufficiently small perturbations in the initial state or parameters, the perturbed trajectory $\boldsymbol{x}(t, \boldsymbol{\lambda})$ (dashed line) remains within the tube for all $t \in [t_0, t_1]$.

References

1. H Khalil, *Nonlinear Systems, 3rd Edit.* (Pearson Education), (2002).
2. R Mehra, On the identification of variances and adaptive kalman filtering. *IEEE Transactions on automatic control* **15**, 175–184 (1970).
3. L Milocco, T Uller, Utilizing developmental dynamics for evolutionary prediction and control. *Proc. Natl. Acad. Sci.* **121**, e2320413121 (2024).
4. KJ Åström, B Wittenmark, *Computer-controlled systems: theory and design.* (Courier Corporation), (2013).
